# Risk of herpes zoster in patients prescribed inhaled corticosteroids: a cohort study

**DOI:** 10.1186/1471-2466-11-59

**Published:** 2011-12-16

**Authors:** Pierre Ernst, Sophie Dell'Aniello, Yann Mikaeloff, Samy Suissa

**Affiliations:** 1 From the Center for Clinical Epidemiology, Lady Davis Research Institute, Jewish General Hospital, Montreal, Canada (PE, SD, SS) and Service de Neuropédiatrie, Faculté de Médecine Paris-Sud 11, France (YM

**Keywords:** herpes zoster, inhaled corticosteroids, adverse drug effects, observational studies, cytochrome P450

## Abstract

**Background:**

Little is known concerning risk factors for herpes zoster in the general population. We hypothesised that inhaled corticosteroids (ICS) are a risk factor for herpes zoster especially among users of inhibitors of cytochrome P450 enzymes involved in their metabolism.

**Methods:**

We identified a cohort of adult users of respiratory medications in the General Practice Research Database and carried out a nested case control analysis of inhaled corticosteroid use among 8900 new cases of herpes zoster and 88032 controls matching on age and calendar time.

**Results:**

The adjusted odds ratio for the relationship between current use of ICS and the occurrence of herpes zoster was 1.00 (95% confidence interval (CI), 0.94-1.07). There was no increase in risk of herpes zoster even at higher ICS doses; odds ratio 1.05 (95% CI, 0.96-1.14). Among subjects with concomitant prescriptions for an ICS and an inhibitor of cytochrome P450 3A4, the point estimate for the association between herpes zoster and the use of higher doses of inhaled corticosteroids was 1.23 (95% CI, 0.81-1.88).

**Conclusions:**

The use of inhaled corticosteroids, even at high doses and in conjunction with inhibitors of their metabolism, was not a significant risk factor for the occurrence of herpes zoster in adults.

## Background

Inhaled corticosteroids (ICS) are being increasingly used in the treatment of patients with asthma and with chronic obstructive pulmonary disease (COPD). These medications are systemically absorbed and may have systemic adverse effects such as cataracts and fractures [1,2]. The use of high doses of ICS has recently been found to increase the risk of severe pneumonia in patients with COPD [3,4]. Information on other infectious complications of ICS therapy is scant but low doses of oral corticosteroids with similar systemic potency have been linked to an excess of serious infections [5]. Moreover, ICS are metabolised via the cytochrome P450 (CYP) system which is involved in biotransformation of the vast majority of all drugs currently available [6,7]. Therefore patients often find themselves taking several medications competing for the same enzymes required for their biotransformation into inactive compounds which can then be excreted.

Herpes zoster (shingles) results from reactivation of latent varicella zoster virus. The lifetime risk of zoster is estimated to be 10-30% and incidence increases markedly with age, affecting up to 50% of people who live up to 85 years [8,9]. The most common complication of zoster is chronic pain due to post herpetic neuralgia. The risk of this complication can be reduced by the prompt administration of anti-viral agents; however, 20% of people older than 50 years who receive treatment still experience pain six months after rash onset [10]. Factors responsible for the majority of cases occurring in apparently well individuals have not been identified [8].

Given the impairment in cell mediated immunity associated with the use of corticosteroids and the systemic activity of ICS, especially at higher doses, a study of the link between use of ICS and herpes zoster appears warranted. We therefore undertook a study in a large primary care clinical database in order to assess the risk of herpes zoster in relation to use of ICS in a general population of adults and to evaluate whether this risk is increased by the concomitant use of ICS and drugs which inhibit CYP3A4, the enzyme responsible for the hepatic inactivation of corticosteroids.

## Methods

The study cohort was identified from the population-based General Practice Research Database (GPRD), which has been described in detail. It is one of the world's largest computerised databases with longitudinal data, including the demographic characteristics, medical diagnoses, laboratory test data and prescription information for about 6.4 million patients from over 400 General practices in the U.K [11-13]. Subjects were eligible if they were registered as permanent in an up-to-standard practice for at least one year without a diagnosis of herpes and without a prescription for a bronchodilator, a cromolyn, a leukotriene antagonist or an inhaled corticosteroid; in this way, we attempted to include mostly new users of respiratory medications. Subjects then entered the cohort on the day of their third prescription on at least two different dates of a respiratory drug within one year. All cohort members were followed up from cohort entry until a diagnosis compatible with herpes zoster was recorded, death, exit from the practice or December 30^th^, 2007, whichever occurred first. We identified new cases of herpes zoster occurring during follow-up using diagnostic codes previously identified for a study of the risks of NSAIDs for severe skin and soft tissue complications of varicella infections [14]. We carried out a nested case-control analysis where each case was matched to up to 10 controls selected from the cohort, based on age (within 12 months), and presence in the cohort in the year and month (± 30 days) of the diagnosis of herpes zoster in the case.

Inhaled corticosteroid (ICS) exposure included prescriptions for beclometasone, budesonide, triamcinolone, fluticasone and flunisolide, whether dispensed alone or in a combination inhaler with an inhaled β agonist. The estimation of equivalencies were chosen on the basis of relative topical potency and what experts consider to be comparable doses according to the NAEPP expert panel report (EPR-2), figures 3-5b and 3-5c [15] and the Canadian asthma consensus statement, Table 8 [16]. Accordingly, the equivalent doses for inhaled corticosteroids are beclometasone 100 mcg, budesonide 80 mcg, triamcinolone 200 mcg, fluticasone 50 mcg and flunisolide 200 mcg. All doses were converted to fluticasone equivalents and categorised according to defined daily doses [17] of the most recent prescription in the appropriate time window as high (fluticasone 500 mcg per day or more), moderate (fluticasone 200-499 mcg per day), and low (less than 200 mcg per day). We also defined a very high dose category as fluticasone 1000 mcg per day or more. Current use of inhaled corticosteroids was defined as a prescription for an ICS given to the patient within the 60 days before the occurrence of zoster (or the matching index date for controls). Cumulative dose of ICS in the prior year was also calculated and expressed as fluticasone equivalents. Since there are differences in the systemic potencies of the different ICS [18], we also looked at risk by type of ICS.

Statistical analysis was done using conditional logistic regression to calculate the odds ratio of zoster in relation to the use of ICS up to the index date, that is the date the diagnoses of herpes was diagnosed in the case. Crude odds ratios, already adjusted for age and calendar time by matching, were also adjusted for gender and smoking status and for the prescription of oral, nasal and injectable corticosteroids in the same time windows as exposure to ICS. Severity of respiratory disease was accounted for by adjusting for each medication class, including antibiotics, prescribed in the prior year, as well as the occurrence of a hospitalisation with a respiratory diagnosis in the prior year. To adjust for co-morbidity, the presence of cardiovascular disease including hypertension and diabetes were identified by the prescription of cardiovascular and diabetes drugs respectively. We also adjusted for the recording of a diagnosis of cancer (excluding non-melanoma skin cancer) in the prior year as well as a prior history of varicella or zoster. To examine whether a decrease in metabolism of inhaled corticosteroids might increase the risk of herpes zoster, we looked at the relationship with use of inhaled corticosteroids among subjects with concomitant prescriptions of any of the following inhibitors of CYP3A4: amiodarone, aprepipant, clarithromycin, ciprofloxacin, delavirdine, diltiazem, erythromycin, fluoxetine, imatinib, indinavir, isoniazid, itraconazole, ketoconazole, nefazodone, nelfinavir, norfloxacin, ritonavir, saquinavir, tamoxifen, verapamil, and voricovazole.

The study has received ethical approval from The Medicines and Healthcare Products Regulatory Agency (MHRA), protocol 07_047R.

## Results

The cohort consisted of 178,704 adults of whom 57.7% had a diagnosis of asthma recorded, 13.5% a diagnosis of COPD and 23.1% a diagnosis of wheezing noted after cohort entry. The average age at cohort entry was 52.8 years (SD 19.3) and 42.5% of cohort members were men. There were 8,900 cases of herpes zoster identified during cohort follow-up. These were matched to 88,032 controls by age and date of diagnosis. Descriptive information on diagnoses and medication use reported in the prior year is provided in Table 1. Cases of herpes zoster were more commonly women. Monitoring by peak flow (peak flow test %) was common but referral to respiratory specialists was uncommon. The most apparent differences were the greater use of oral corticosteroids and antibiotics in cases than in controls.

**Table 1:** History and Medication Use in the Prior Year among Zoster Cases and Controls

	Cases	Controls
History*	(n = 8900)	(n = 88032)
Age mean (SD)	54.7 (24.4)	54.4 (24.2)
Gender, male %	38.6	44.4
Asthma %	32.5	31.5
Asthma exacerbation %	3.3	2.6
COPD %	10.1	9.6
COPD exacerbation %	2.2	1.7
Wheezing %	5.5	4.9
Other lung disease %	2.8	2.2
Peak Flow test%	30.1	29.2
Referral to respiratory physician %	0.5	0.5
History of varicella %	0.2	0.2
History of zoster %	0.4	0.1
Respiratory Hospitalisation %	1.2	0.9
Cancer %	1.9	1.2
Medications prescribed*		
Inhaled Corticosteroids (ICS) %	47.3	46.6
Beclometasone %	36.3	36.4
Budesonide %	6.9	6.8
Fluticasone %	4.1	3.4
Other ICS %	0.1	0.1
ICS+ Long acting β-agonist %	11.3	9.9
Long acting β-agonist %	7.7	7.2
Short acting β-agonist %	61	60.1
Other respiratory drugs %	27.7	24.6
Nasal corticosteroids %	10.8	10
Injection corticosteroids %	1.9	1.6
Oral corticosteroids %	19.9	15.2
Antibiotics %	61.5	52.6
Anti-diabetics drugs %	5.6	5
Cardiac drugs %	46.4	42.9

The adjusted odds ratio for the relationship between current use of ICS and the occurrence of herpes zoster was 1.00 (95% confidence interval (CI), 0.94-1.07). In this same analysis, the risk associated with the prescription of oral corticosteroids was significantly elevated; adjusted odds ratio 1.23 (95% CI, 1.15-1.31). As shown in Table 2, there is no increase in risk of herpes zoster at higher ICS doses; adjusted odds ratio 1.05 (95% CI, 0.96-1.14). Even when looking at very high doses equivalent to fluticasone 1000 mcg per day or more, there was no significant risk demonstrable; adjusted odds ratio 0.93 (95% CI, 0.78-1.12). We looked at whether cumulative dose of inhaled corticosteroids over the prior 12 months was associated with risk of zoster. The risk did not increase with increasing exposure such that even among subjects prescribed more than 4 inhalers of high dose inhaled corticosteroid (fluticasone 500 mcg per day or more) in the past year the adjusted odds ratio was 0.97 (95% CI, 0.89-1.07).

**Table 2:** Inhaled Corticosteroid (ICS) use and risk of Herpes Zoster in 8900 cases and 88032 controls

	Cases	Controls	Crude odds ratio	Adjusted odds ratio* (95% CI†)
**ICS**				
no use%	45.4	46.9	1.00	Reference
high dose%	13.1	11.6	1.19	1.05 (0.96-1.14)
medium dose%	14.3	14.7	1.01	0.97 (0.90-1.05)
low dose%	2.9	2.9	1.05	1.02 (0.89-1.17)
				

*Adjusted for gender, smoking status, all the variables in Table 1, as well as past use of ICS.

†CI: confidence interval

We examined the effect of different corticosteroids and were unable to identify an excess risk with any particular ICS; adjusted odds ratio for fluticasone 1.03 (95% CI, 0.93-1.15). Table 3 shows the risk of herpes zoster among subjects with concomitant prescriptions for an ICS and an inhibitor of CYP3A4. The number of cases was substantially less (n = 475) due to the inability to find appropriate matched controls for most cases. While the point estimate for the odds ratio was elevated at 1.24 among those prescribed higher doses of inhaled corticosteroids, the confidence interval was wide (95% CI, 0.81-1.90) and included no association.

**Table 3:** Inhaled Corticosteroids (ICS) and the Risk of Herpes Zoster Among Cases and Controls Treated with Inhibitors of Cytochrome Oxidase P450 3A4*

	Cases (n = 475)	Controls (n = 760)	Crude Odds Ratio	Adjusted Odds Ratio† (95% CI ‡)
**ICS**				
no use%	35.6	36.1	1.0	reference
high dose%	24.4	20.8	1.13	1.24 (0.81-1.90)
medium dose%	16.8	18.2	0.95	0.94 (0.63-1.41)
low dose%	2.5	3.3	0.75	0.66 (0.29-1.48)

*see list of inhibitors of cytochrome P450 3A4 in methods.

†Adjusted for gender, smoking status, all the variables in Table 1, as well as past use of ICS.

‡CI: confidence interval

## Discussion

While our results strongly suggest that inhaled corticosteroids do not represent a significant risk for zoster in adults, this lack of an association might possibly to due to methodological problems such as insufficient sample size and misclassification of outcome or of exposure. Insufficient sample size is unlikely to explain the overall results given the 8900 cases of zoster and the frequent prescribing of inhaled corticosteroids in our cohort. As for the quality of the information on diagnoses and prescription in the GPRD, this has been repeatedly demonstrated [11,19]. We cannot, however, rule out the possibility that the lack of an effect results from patients not taking the inhaled corticosteroids they were prescribed. An association between use of inhaled corticosteroids and risk of herpes zoster might be masked if physicians were less likely to prescribe inhaled corticosteroids in patients thought to at increased risk of zoster. This is unlikely to explain the lack of an association found here since we were able to adjust for known and suspected risk factors for zoster present in the year prior to diagnosis and that information on these risk factors and on prescribed medication was gathered prospectively. Furthermore we included mostly new users of respiratory medications such that subjects at highest risk of the outcome had not ceased exposure to inhaled corticosteroids [20]. Subjects prescribed inhaled corticosteroids, especially at higher doses are also likely to be older, sicker and to have been prescribed oral corticosteroids and therefore at greater risk of zoster [21]. This explains the slight increases seen in the crude odds ratios of the association of higher doses of inhaled corticosteroids with zoster. The lower odds ratios after adjustment strongly suggest that there is no additional risk associated with the use of inhaled corticosteroids. The 60 day time window for current exposure is based on our previous study of severe pneumonia which found risk for this infectious complication to be maximal for ICS prescribed within the prior 60 days and for the effect to wane quickly thereafter [4]. Even if ICS are most commonly dispensed as a 30 day supply, based on our prior experience, there is sufficient variability in prescribing and in patient compliance with the prescribed dose to expect that current exposure should include a 30 day period after the nominal termination of a dispensed prescription. To examine the possibility that an association may have been missed due to inappropriate timing of the exposure window and the possibility that excess risk may be associated with cumulative rather than recent exposure, we also examined the risk of zoster in relation to the total dose of inhaled corticosteroids prescribed in the past year. Again, no risk was apparent in the highest dose category (fluticasone 500 mcg per day or more) even among patients making regular use of these medications (more than four prescriptions in the past year). It must be noted that the analysis examining the risk of zoster among concomitant users of inhaled corticosteroids and inhibitors of pertinent cytochrome oxidase enzymes had limited power since matched controls could only be identified for 475 cases of zoster. Thus, we cannot rule out an excess risk of zoster in patients making concomitant use of ICS and potent inhibitors of CYP3A4 such as ritonavir. Yang and colleagues recently reported an excess in risk of herpes zoster in patients with COPD [22]. Such patients comprise approximately 10% of our study population. They also found a greater risk if the COPD patients had received oral corticosteroids while the risk was not significantly elevated in COPD patients receiving inhaled corticosteroids as compared to those not prescribed any corticosteroids.

## Conclusion

We identified 8900 cases of herpes zoster in a large cohort of patients followed by general practitioners in the U.K. After adjusting for known (age, gender, oral corticosteroids, malignancy [9] and potential risk factors such as type of chronic respiratory disease [21,22] and diabetes [23], there was no excess in risk of herpes zoster among patients prescribed inhaled corticosteroids, even at high doses, nor among patients prescribed concomitant inhibitors of corticosteroid metabolism which would be expected to increase the systemic adverse effects of inhaled corticosteroids.

Factors responsible for the more frequent cases of zoster occurring in individuals without marked impairment in their immune status are unknown. Such lack of knowledge has been called surprising given the frequency of the problem and its significant morbidity [8]. We had hypothesized that use of inhaled corticosteroids, especially at higher doses and in combination with inhibitors of pertinent cytochrome oxidase enzymes, might be a risk for zoster; however, we were unable to find any evidence of an excess risk of zoster with the use of inhaled corticosteroids.

## List of abbreviations

CYP: cytochrome P450 enzymes; ICS: inhaled corticosteroids; COPD: Chronic Obstructive Pulmonary Disease; GPRD: General Practice Research Database; U.K.: United Kingdom; NSAIDs: non-steroidal anti-inflammatory drugs; NAEP: National Asthma Expert Panel; SD: standard deviation; CI: confidence interval; mcg: micrograms.

## Competing interests

Pierre Ernst has received speaker fees, and/or has served on advisory boards for AstraZeneca, Boeringher-Ingelheim, GlaxoSmithKline, Merck, Novartis, Nycomed and Pfizer. Samy Suissa has received speaker fees, research grants and/or has served on advisory boards for AstraZeneca, Boeringher-Ingelheim, GlaxoSmithKline, Merck, Novartis and Pfizer. Sophie Dell'Aniello and Yann Mikaeloff have no conflicts of interest to declare.

## Authors' contributions

PE came up with the question and wrote the manuscript. YM provided the codes to identify cases of Herpes. SD and SS were responsible for data analysis. All authors were involved in interpreting the data. All authors have read and approved the final manuscript.

## Pre-publication history

The pre-publication history for this paper can be accessed here:

http://www.biomedcentral.com/1471-2466/11/59/prepub
